# Supplementary material for: Silencing of TRAF5 enhances necroptosis in hepatocellular carcinoma by inhibiting LTBR-mediated NF-κB signaling
Source: PeerJ. 2023 Jun 22;11:e15551. doi: 10.7717/peerj.15551 (PMC10290833; doi:10.7717/peerj.15551)

Figure 1B

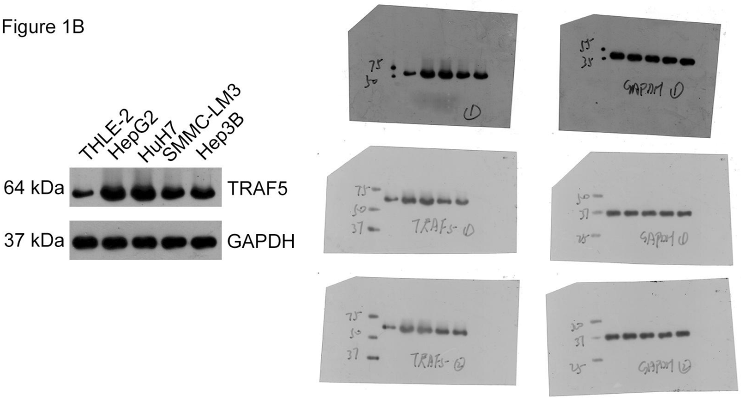

Figure 2B

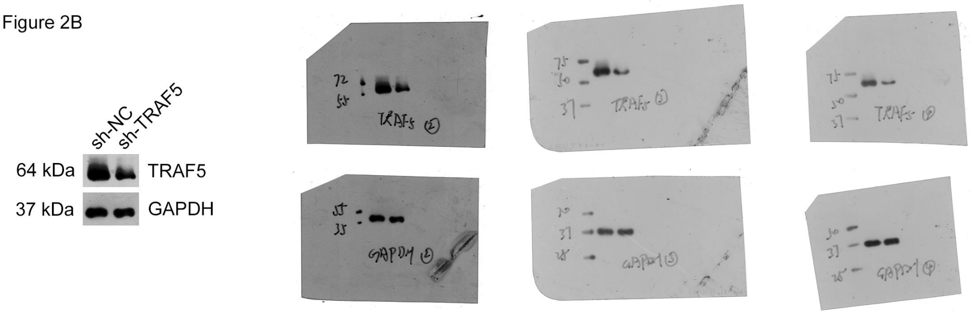

Figure 2G

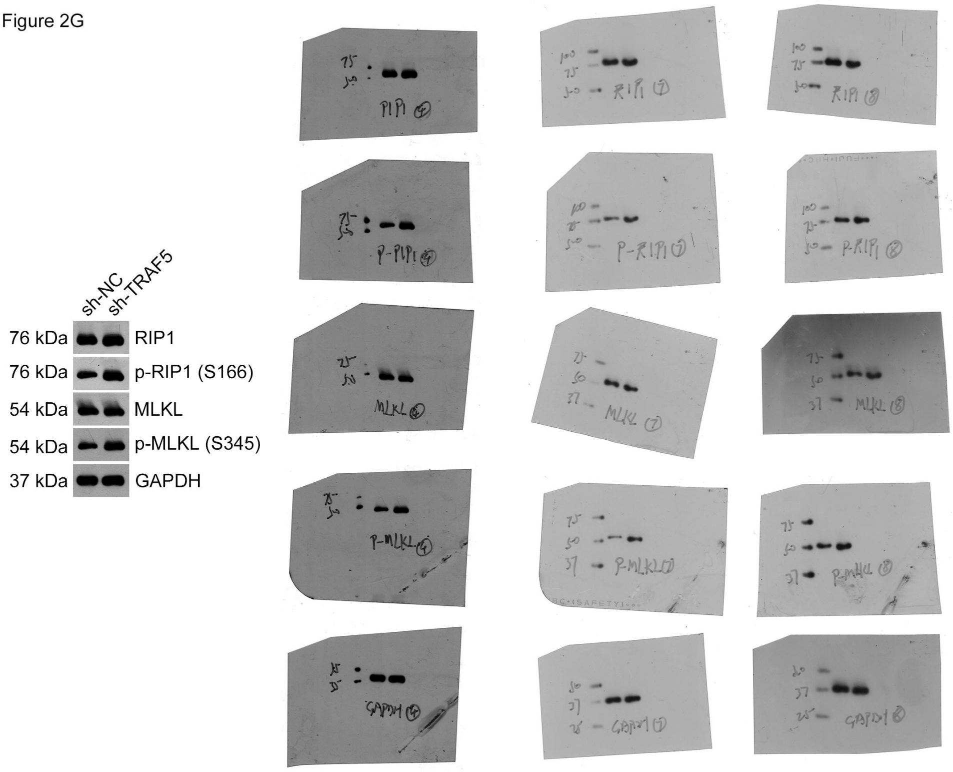

Figure 3B

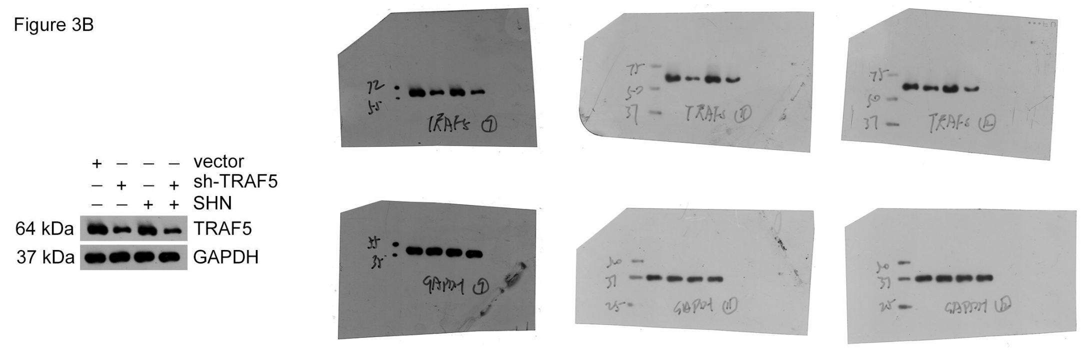

Figure 3F

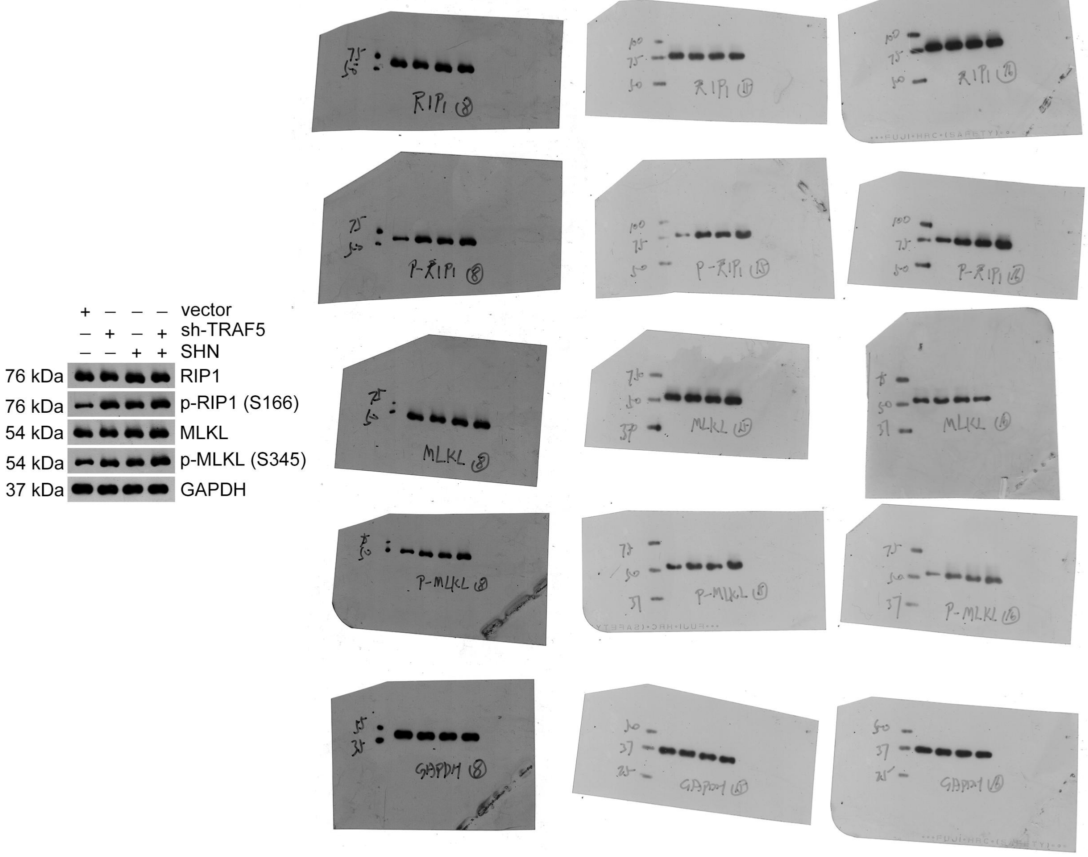

Figure 4A

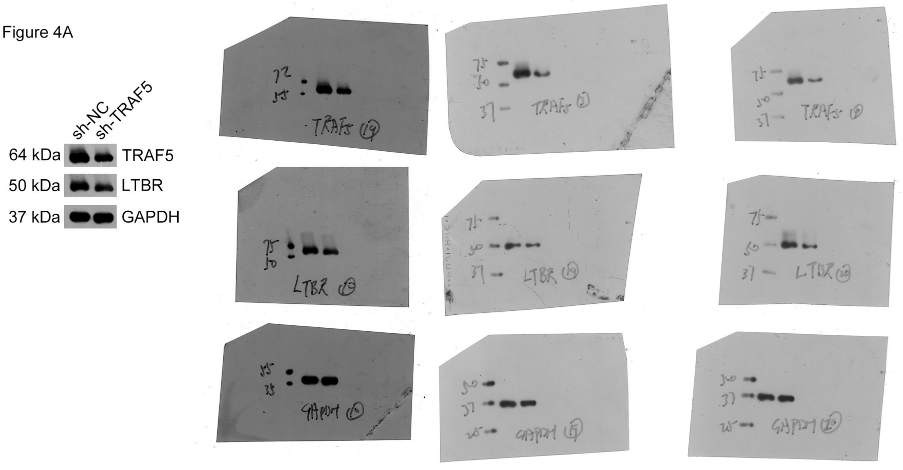

Figure 4B

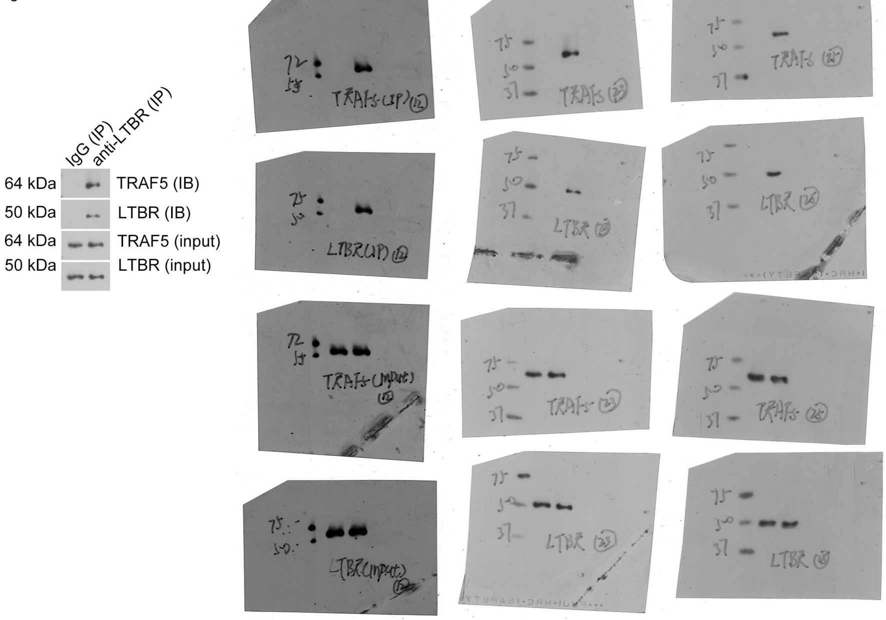

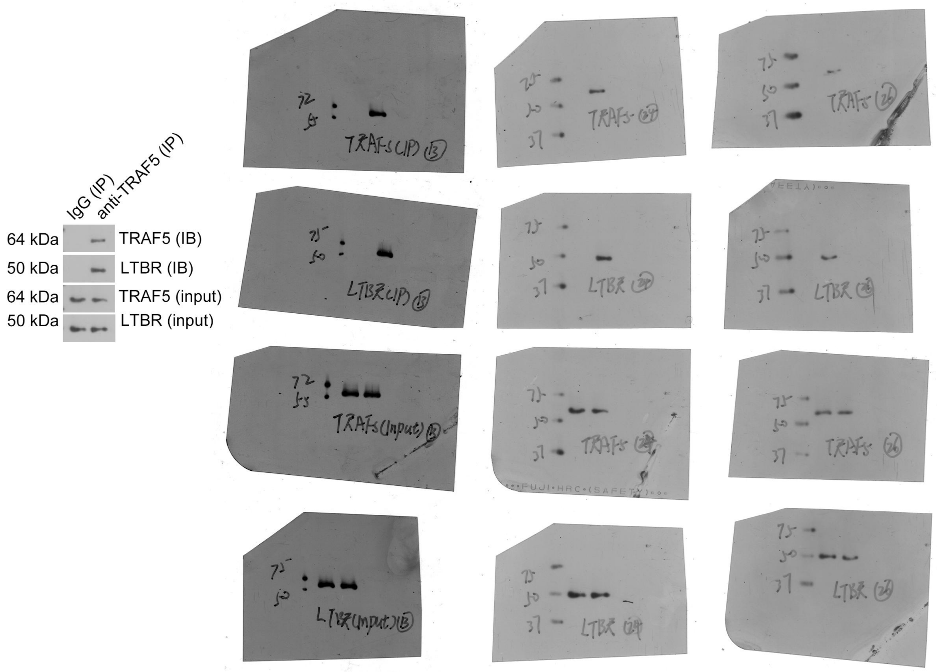

Figure 5B

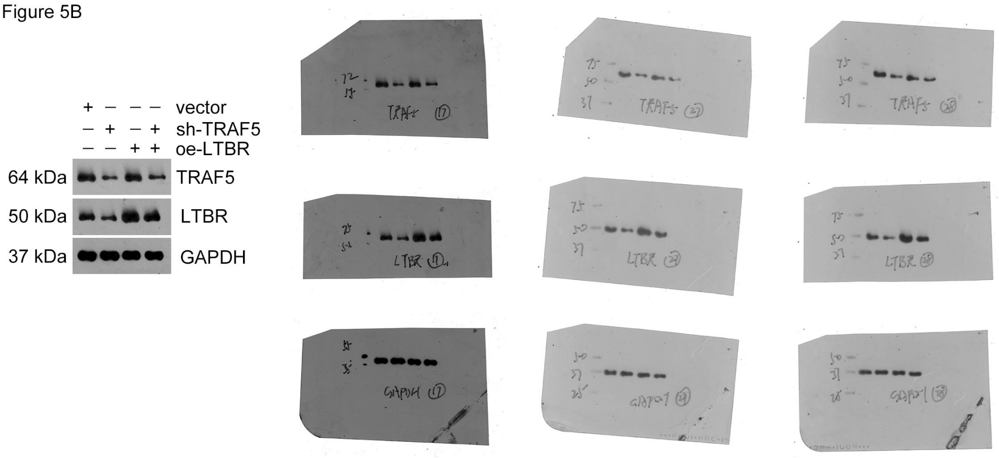

Figure 5E

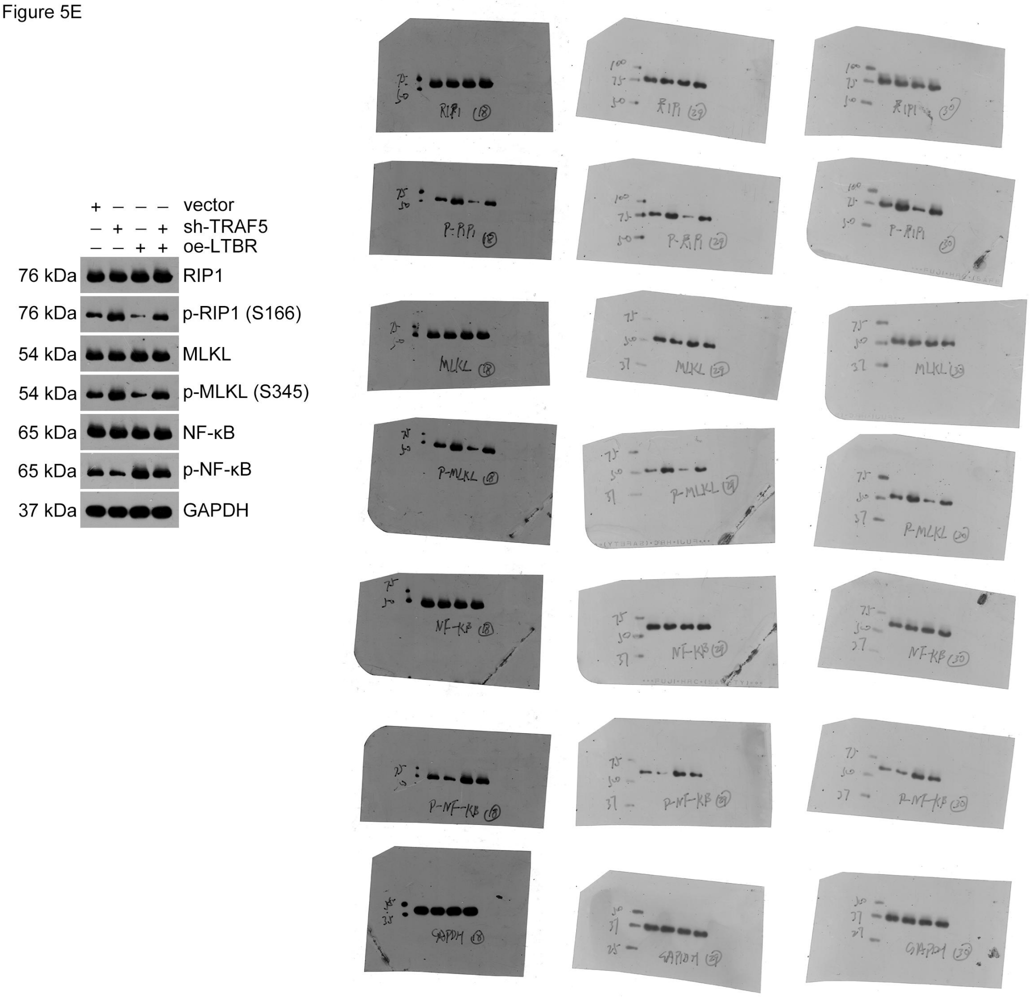

Figure 6A

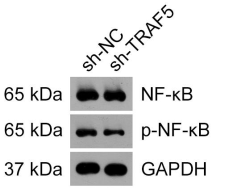

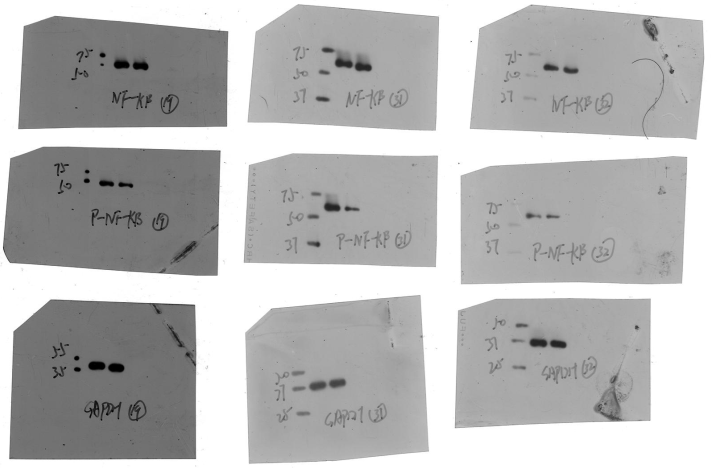

Figure 6C

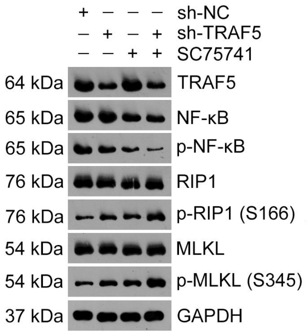

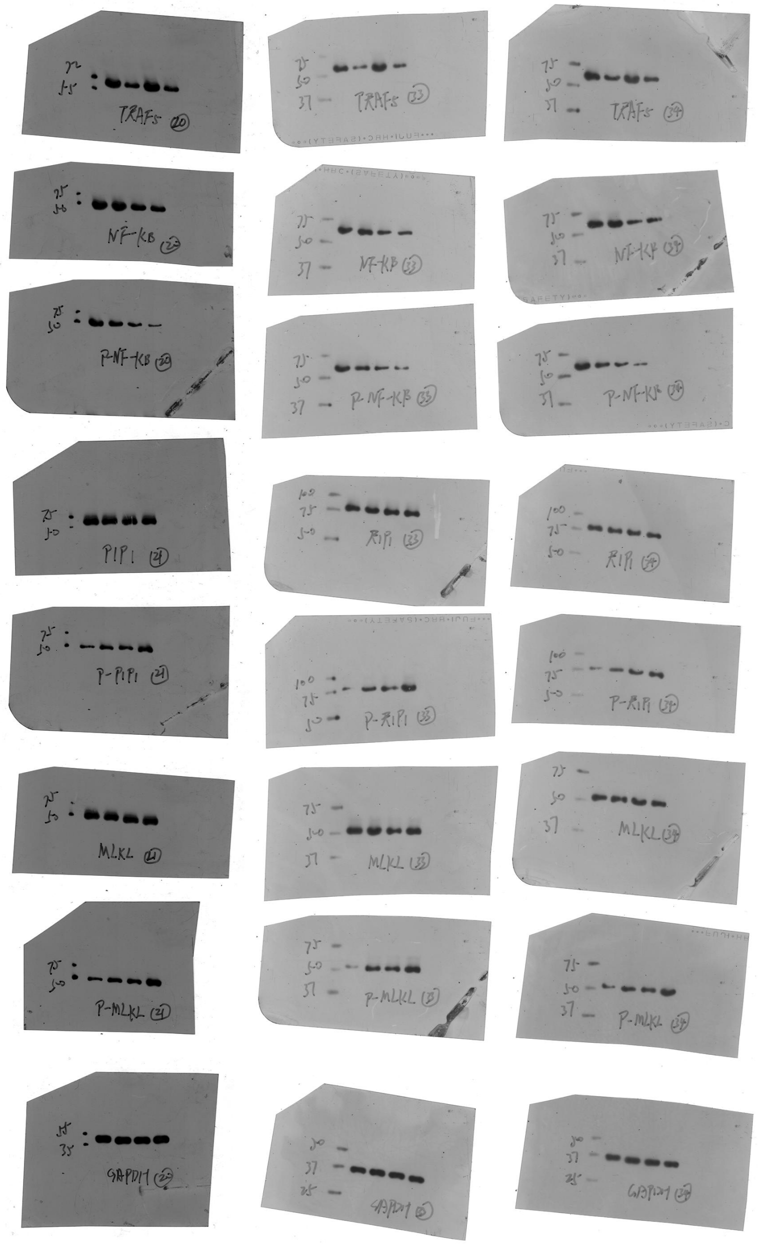

Figure 7D

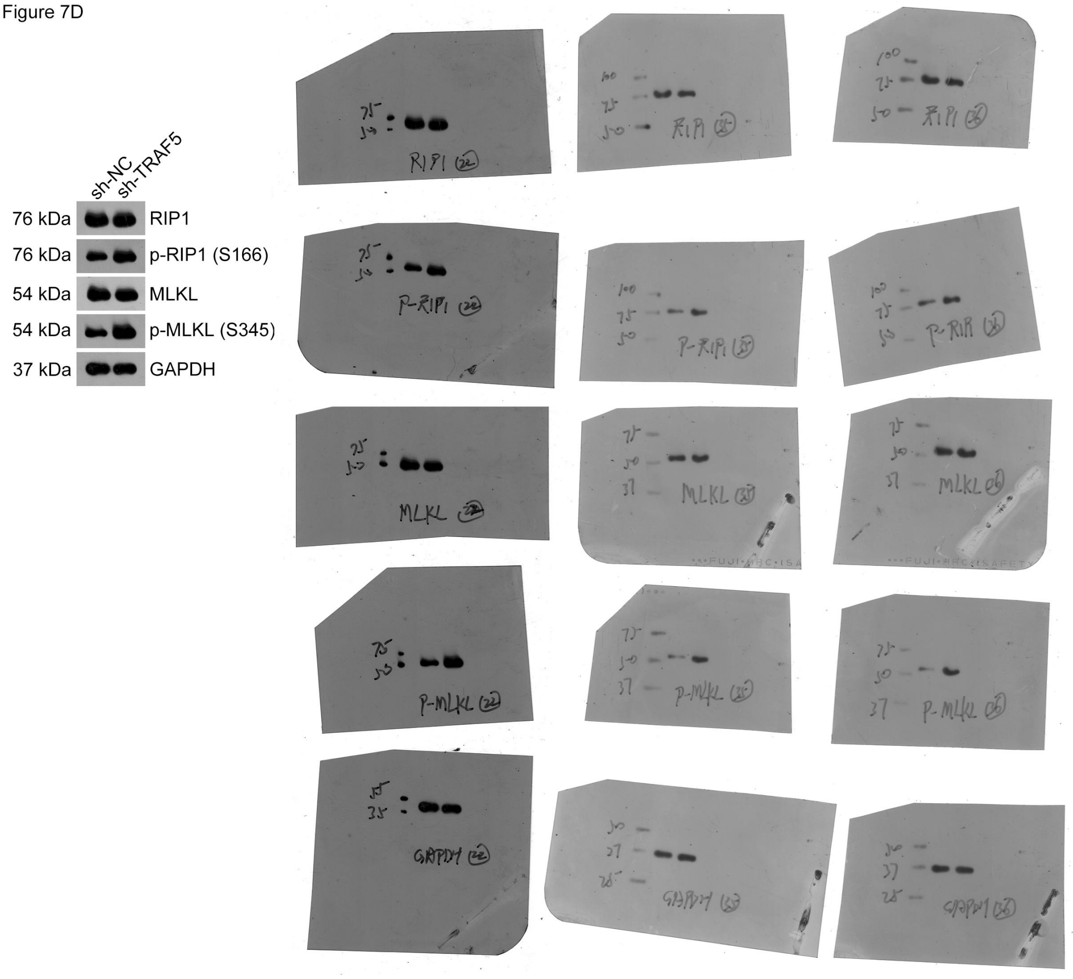
sh-NC
sh-TRAF5
76 kDa
RIP1
76 kDa
p-RIP1 (S166)
54 kDa
MLKL
54 kDa
p-MLKL (S345)
37 kDa
GAPDH

Figure S2B

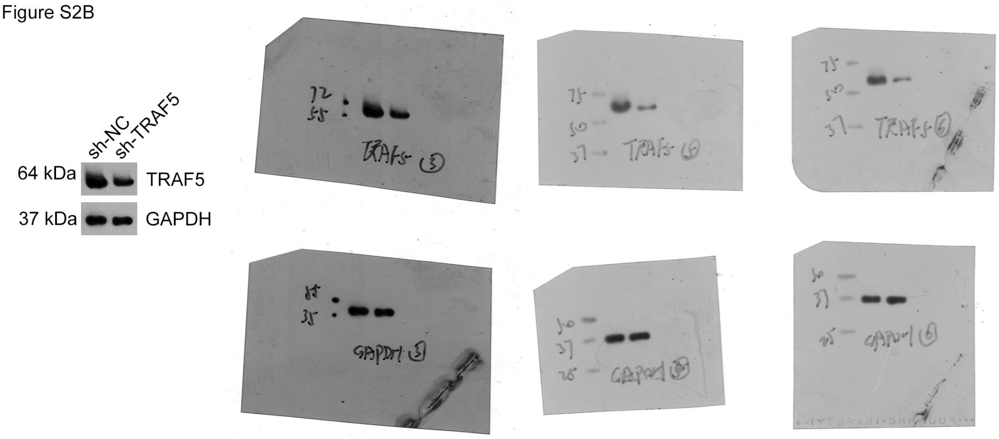

Figure S2G

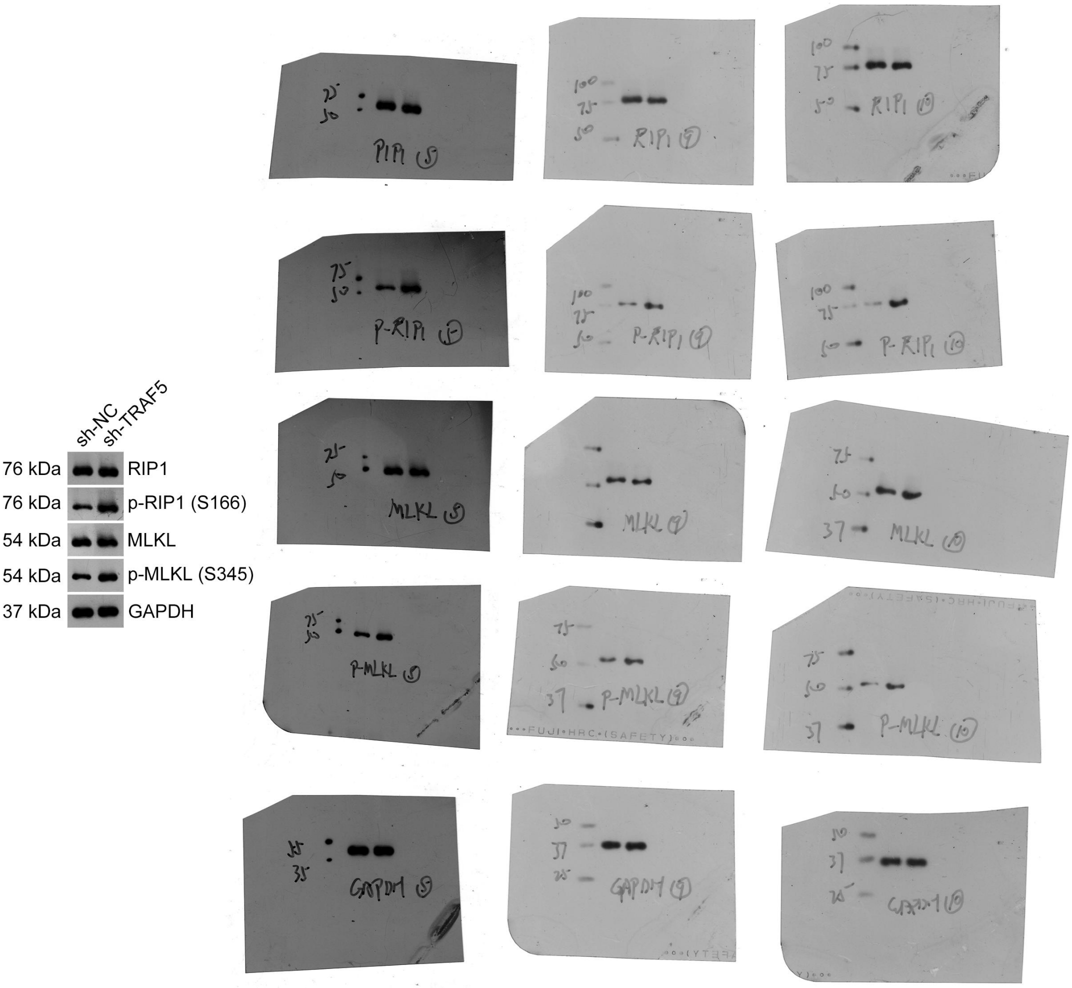

Figure S3B

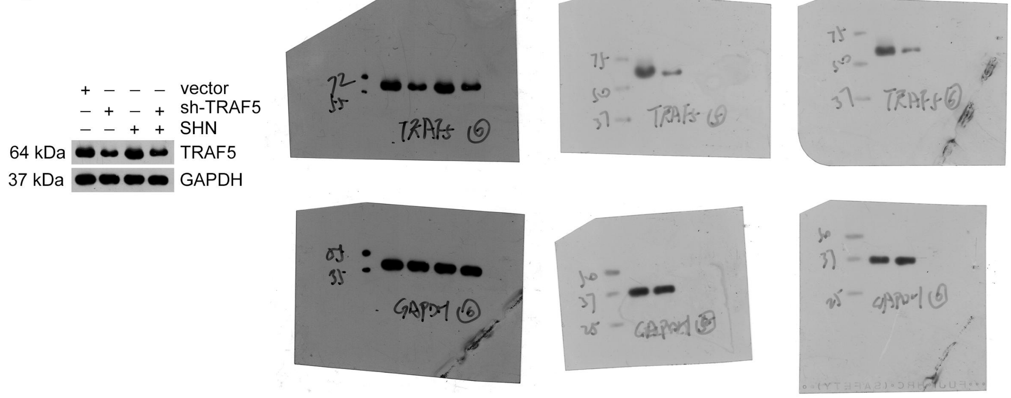

Figure S3F

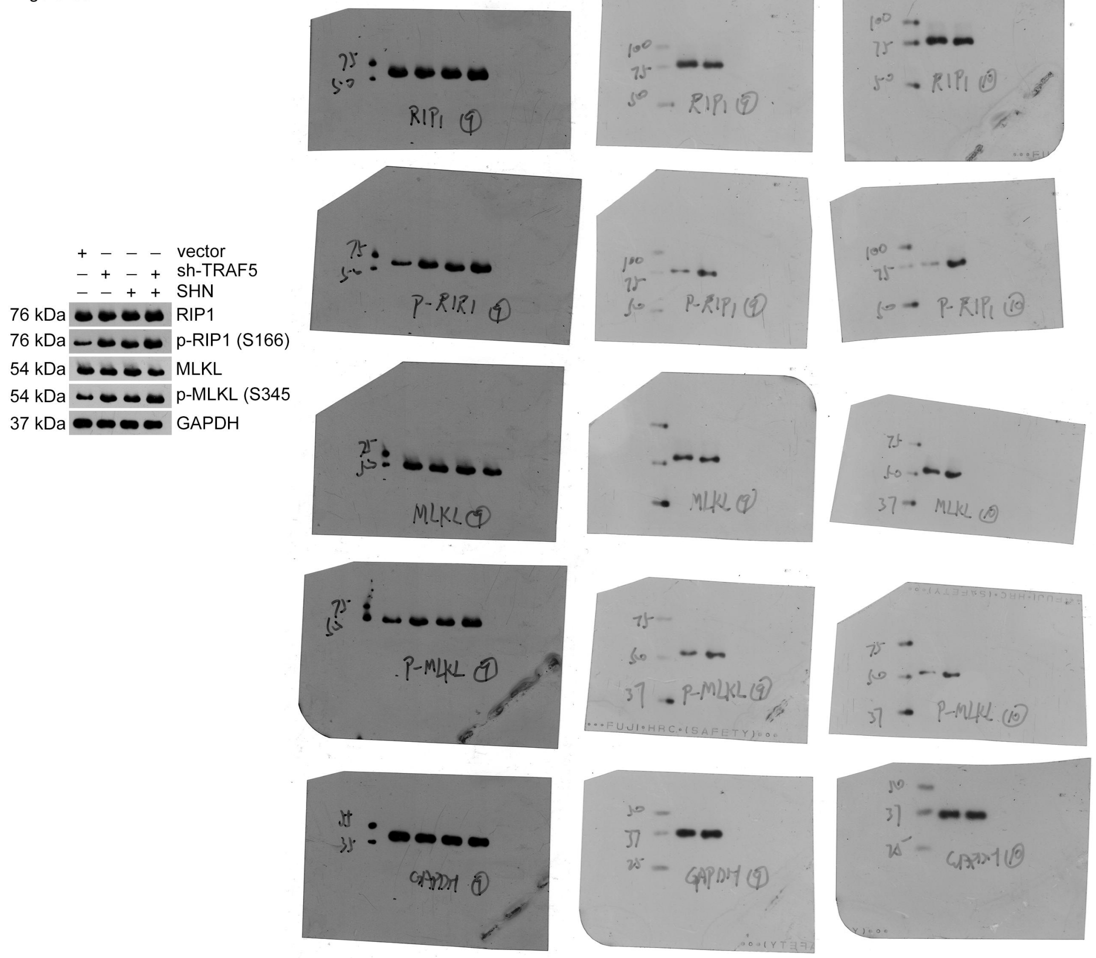

Supplement: Supplemental Information 1 [file peerj-11-15551-s001.pdf]
