# Supplementary figures and images for: Silencing of TRAF5 enhances necroptosis in hepatocellular carcinoma by inhibiting LTBR-mediated NF-κB signaling
Source: PeerJ. 2023 Jun 22;11:e15551. doi: 10.7717/peerj.15551 (PMC10290833; doi:10.7717/peerj.15551)

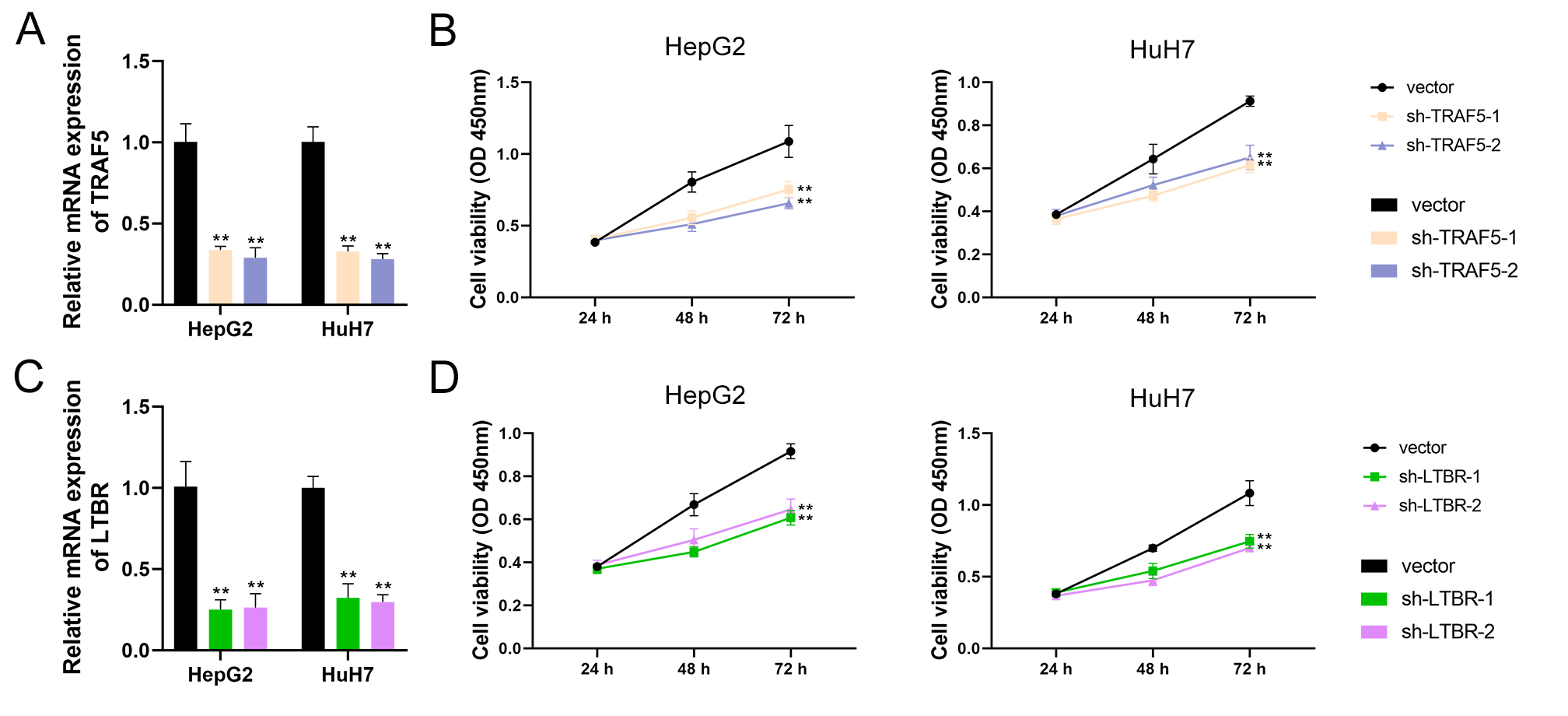

Supplement: Supplemental Information 2 — (A) Detection of TRAF5 mRNA expression in HepG2 and HuH7 using qRT-PCR. (B) Detection of the viability of HepG2 and HuH7 cells using CCK-8. (C) Detection of LTBR mRNA expression in HepG2 and HuH7 using qRT-PCR. (D) Detection of the viability of HepG2 and HuH7 cells using CCK-8. Data were expressed as mean ± standard deviation. **P < 0.01 vs vector group. TRAF5, TNF receptor-associated factor 5; LTBR, lymphotoxin beta receptor; HCC, hepatocellular carcinoma; qRT-PCR, quantitative real-time polymerase chain reaction; CCK-8, cell counting kit-8. [file peerj-11-15551-s002.tif]

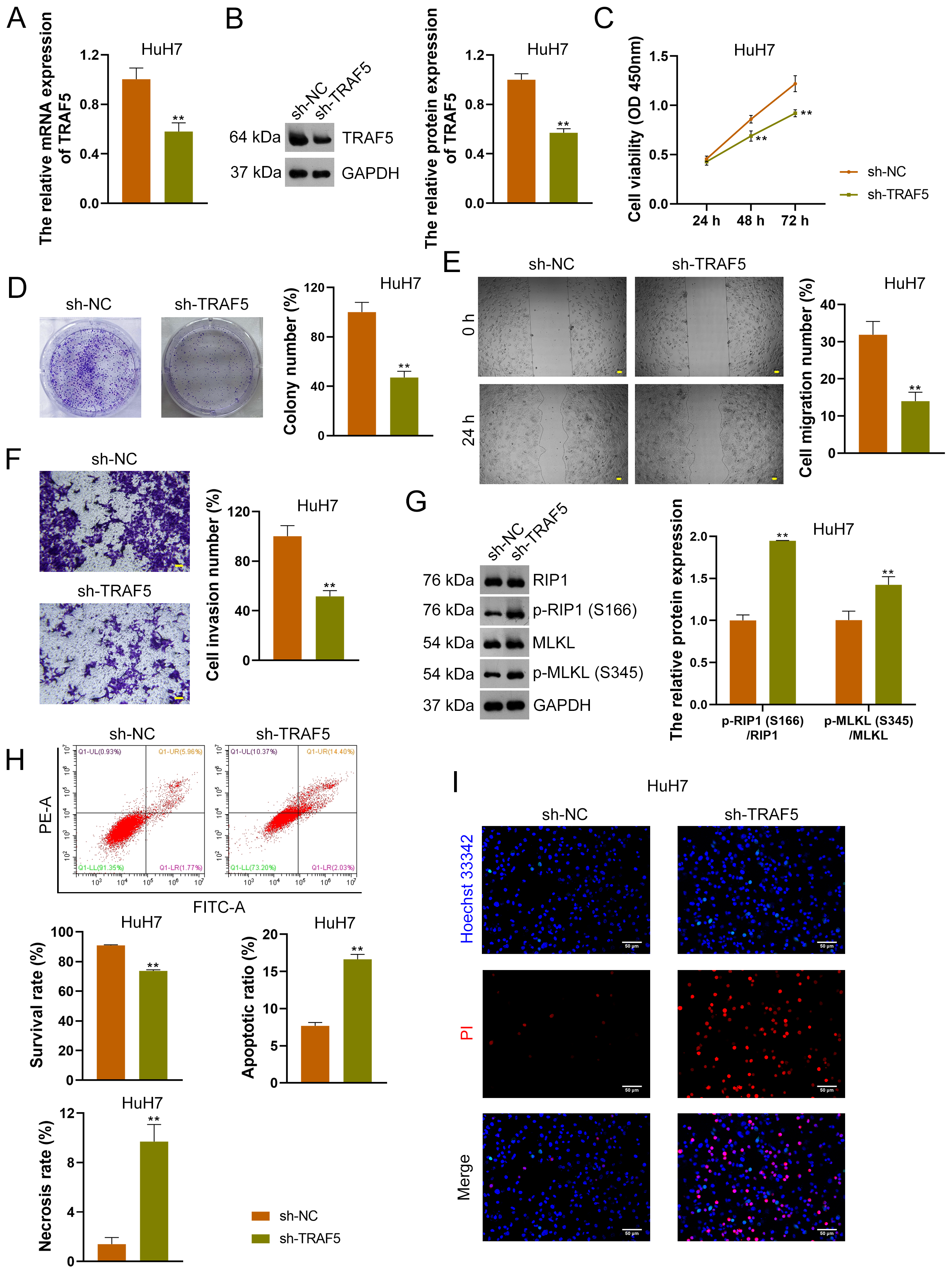

Supplement: Supplemental Information 3 — (A and B) Detection of TRAF5 expression in HuH7 cells using qRT-PCR and western blotting. (C) Detection of the viability of HuH7 cells using CCK-8. (D) Detection of the colony formation of HuH7 cells. (E) Detection of the migration of HuH7 using cell wound healing assay (Scale bar = 50 μm). (F) Detection of the invasion of HuH7 cells using Transwell assay (Scale bar = 50 μm). (G) Detection of the protein expression of p-RIP1 (S166)/RIP1 and p-MLKL (S345)/MLKL in HuH7 cells using western blotting. (H) Detection of the survival, apoptotic, and necrosis ratios of HuH7 cells using flow cytometry. (I) Detection of the necrosis and apoptosis of HuH7 cells using Hoechst 33342/PI double-staining. Scale bar = 50 μm. Data were expressed as mean ± standard deviation. **P < 0.01 vs sh-NC group. TRAF5, TNF receptor-associated factor 5; HCC, hepatocellular carcinoma; qRT-PCR, quantitative real-time polymerase chain reaction; CCK-8, cell counting kit-8; RIP1 receptor-interacting protein 1; MLKL, mixed lineage kinase domain-like; PI, propidium iodide. [file peerj-11-15551-s003.tif]

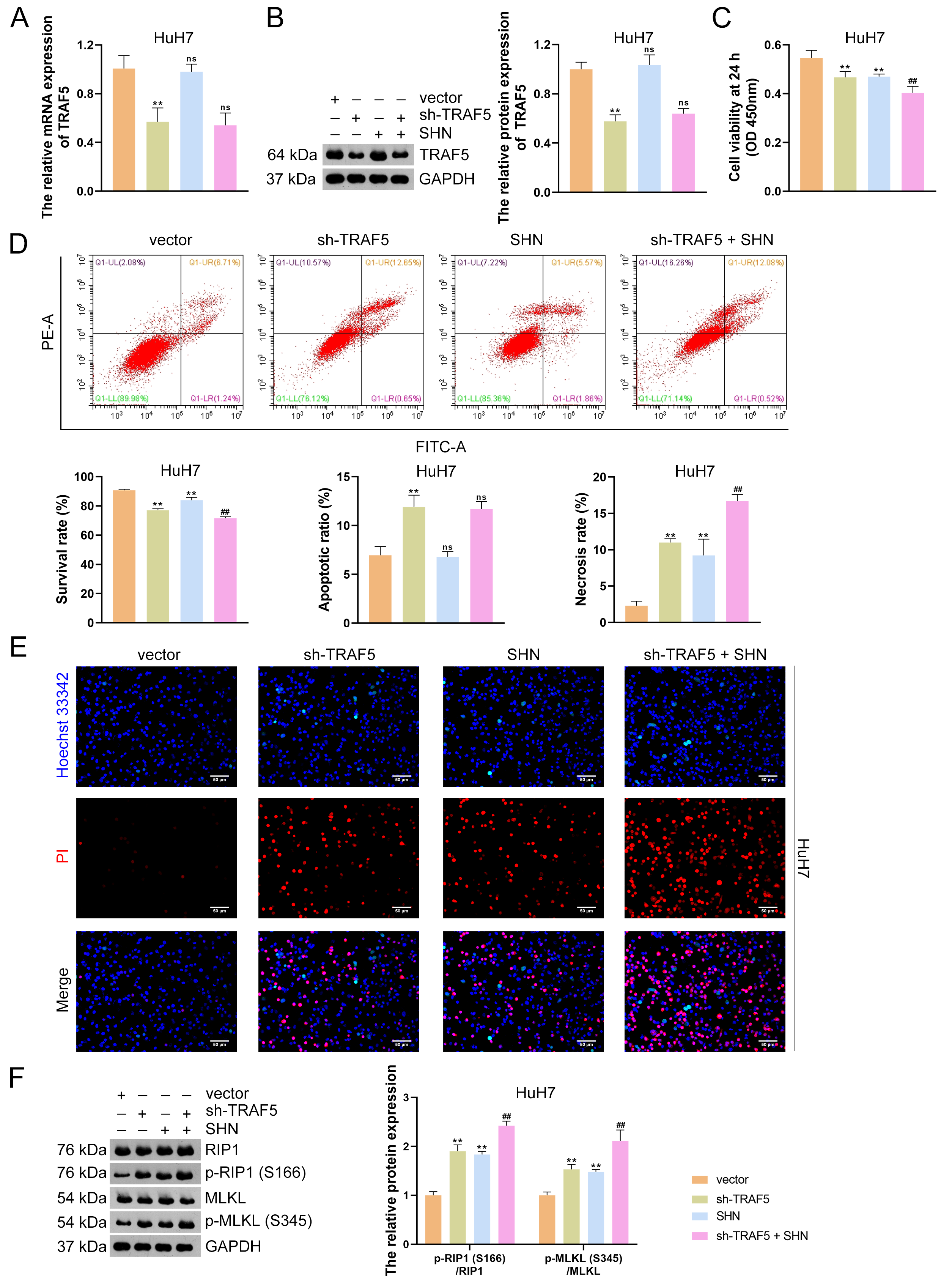

Supplement: Supplemental Information 4 — (A and B) Detection of TRAF5 expression in HuH7 cells using qRT-PCR and western blotting. (C) Detection of the viability of HuH7 cells using CCK-8. (D) Detection of the survival, apoptotic, and necrosis ratios of HuH7 cells using flow cytometry. (E) Detection of the necrosis and apoptosis of HuH7 cells using Hoechst 33342/PI double-staining. Scale bar = 50 μm. (F) Detection of the protein expression of p-RIP1 (S166)/RIP1 and p-MLKL (S345)/MLKL in HuH7 cells using western blotting. Data were expressed as mean ± standard deviation. **P < 0.01 vs vector group; ##P < 0.01 vs sh-TRAF5 group; ns denotes no statistical significance between groups. TRAF5, TNF receptor-associated factor 5; HCC, hepatocellular carcinoma; qRT-PCR, quantitative real-time polymerase chain reaction; CCK-8, cell counting kit-8; RIP1 receptor-interacting protein 1; MLKL, mixed lineage kinase domain-like; PI, propidium iodide. [file peerj-11-15551-s004.tif]
